# Laparoscopic Roux-en-Y Duodenojejunostomy for Duodenal Obstruction after Endovascular Aneurysm Repair Mimicking Superior Mesenteric Artery Syndrome: A Case Report

**DOI:** 10.70352/scrj.cr.26-0144

**Published:** 2026-06-04

**Authors:** Ryusei Takahashi, Yoshihiko Kakiuchi, Yudai Mimata, Shunya Hanzawa, Nobuhiko Kanaya, Hajime Kashima, Satoru Kikuchi, Yoshitaka Kondo, Shinji Kuroda, Shunsuke Kagawa, Toshiyoshi Fujiwara

**Affiliations:** Department of Gastroenterological Surgery, Okayama University Graduate School of Medicine, Dentistry and Pharmaceutical Sciences, Okayama, Okayama, Japan

**Keywords:** superior mesenteric artery syndrome, blind loop syndrome, Roux-en-Y duodenojejunostomy

## Abstract

**INTRODUCTION:**

Superior mesenteric artery (SMA) syndrome is a rare cause of duodenal obstruction, typically associated with weight loss–related narrowing of the aortomesenteric angle. In contrast, fixed mechanical compression after endovascular aneurysm repair (EVAR) may produce duodenal obstruction that mimics SMA syndrome through a different mechanism.

**CASE PRESENTATION:**

A 79-year-old man developed recurrent postprandial abdominal pain and vomiting 4 years after EVAR. CT performed during symptomatic episodes showed marked compression of the horizontal duodenum by the aneurysm sac, whereas the aortomesenteric angle and distance were preserved during asymptomatic periods. Based on these findings, the patient was diagnosed with a duodenal obstruction after EVAR mimicking SMA syndrome. Because conservative treatment was ineffective, a laparoscopic duodenojejunostomy with Roux-en-Y reconstruction was performed to bypass the obstruction and prevent blind loop formation. The postoperative course was uneventful, and no symptom recurrence or complications were observed during 2 years of follow-up.

**CONCLUSIONS:**

Laparoscopic duodenojejunostomy with Roux-en-Y reconstruction may be a safe and effective treatment option for SMA syndrome–like duodenal obstruction caused by EVAR.

## Abbreviations

AAA
: abdominal aortic aneurysm
BLS
: blind loop syndrome
EVAR
: endovascular aneurysm repair
SMA
: superior mesenteric artery

## INTRODUCTION

SMA syndrome is a rare condition characterized by narrowing of the lumen of the horizontal portion of the duodenum due to compression between the abdominal aorta and the SMA, resulting in impaired duodenal passage. It is commonly associated with significant weight loss and reduction of retroperitoneal fat; however, anatomical changes related to AAA have also been recognized as an important contributing factor. In recent years, several reports have suggested that increased aneurysm sac pressure or endoleaks after EVAR may lead to duodenal compression and the development of SMA syndrome–like symptoms.^1)^ However, such cases remain extremely rare. While duodenojejunostomy is widely performed as a surgical treatment for SMA syndrome,^2)^ cases of suspected SMA syndrome–like compression after EVAR have generally been managed with conservative treatment or vascular interventions, and reports describing surgical gastrointestinal bypass are scarce. From a long-term perspective, duodenojejunostomy is associated with a potential risk of BLS, which remains an important concern, particularly in patients with persistent anatomical or mechanical compression.

Here, we report a case of duodenal obstruction mimicking SMA syndrome that resulted from fixed mechanical compression after EVAR, a mechanism distinct from that of classic SMA syndrome. The patient was successfully treated with laparoscopic duodenojejunostomy with Roux-en-Y reconstruction, performed prophylactically to prevent blind loop formation. We also discuss the clinical significance of this surgical approach with reference to the relevant literature.

## CASE PRESENTATION

A 79-year-old man, with a height of 1.65 m, a weight of 66.3 kg, and a body mass index of 24.2 kg/m^2^, presented to a previous hospital with recurrent episodes of abdominal pain and vomiting. He had no recent weight loss or gain. Non-contrast abdominal CT performed during symptomatic episodes revealed marked narrowing of the horizontal portion of the duodenum between the abdominal aorta and the SMA, with a severely reduced aortomesenteric distance of approximately 3 mm (**Fig. 1**). These symptoms began 4 years after EVAR for an AAA and occurred predominantly after meals. Conservative management was repeatedly attempted but provided only temporary relief. In contrast, contrast-enhanced CT performed during asymptomatic periods demonstrated a preserved aortomesenteric angle of approximately 70° and an aortomesenteric distance of 18 mm at the level of the horizontal portion of the duodenum (**Fig. 2**). Although these findings did not meet the anatomical criteria for classic SMA syndrome, the patient was considered to have duodenal obstruction caused by physical compression following EVAR, representing an SMA syndrome–like condition. Based on the consistency between symptoms and imaging findings, surgical intervention analogous to that for SMA syndrome was deemed necessary. Considering both safety and efficacy, as well as the need to prevent postoperative BLS, laparoscopic duodenojejunostomy with Roux-en-Y reconstruction was selected.

**Fig. 1:**
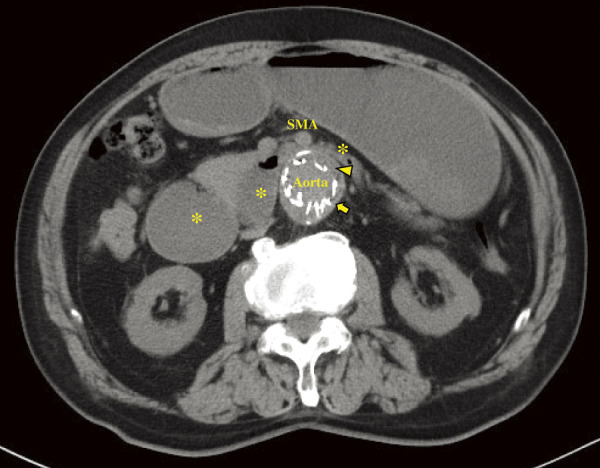
Non-contrast abdominal CT image obtained during the symptomatic period. The arrowhead indicates the aneurysm sac, the arrow indicates the stent graft, and the asteriks indicate the duodenum. SMA, superior mesenteric artery

**Fig. 2:**
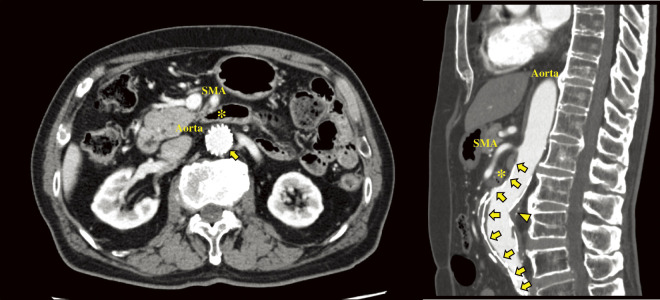
Contrast-enhanced abdominal CT image obtained during the asymptomatic period. The arrowhead indicates the aneurysm sac, the arrows indicate the stent graft, and the asterisk indicates the duodenum. SMA, superior mesenteric artery

The operation was performed under general anesthesia with the patient in the supine position. Four ports were placed, and the horizontal and descending portions of the duodenum were exposed. The jejunum was divided approximately 15 cm distal to the ligament of Treitz, and selected mesenteric vessels were divided as needed to ensure adequate mobility of the distal jejunal limb. A small enterotomy was made in the horizontal portion of the duodenum, and a side-to-side duodenojejunostomy was created using a linear stapler (**Fig. 3A**). The common entry hole was closed with a 2-layer, hand-sewn suture. Subsequently, a Roux-en-Y limb was constructed using the proximal jejunum, and a side-to-side jejunojejunostomy was performed (**Fig. 3B**). Mesenteric defects were closed, and a drain was placed near the anastomosis before completion of the procedure. The operative time was 162 min, and blood loss was minimal. Oral intake was resumed on POD 5, the drain was removed on POD 7, and the patient was discharged on POD 9. At 2 years of follow-up, the patient remains asymptomatic, with no postoperative complications, including BLS.

**Fig. 3:**
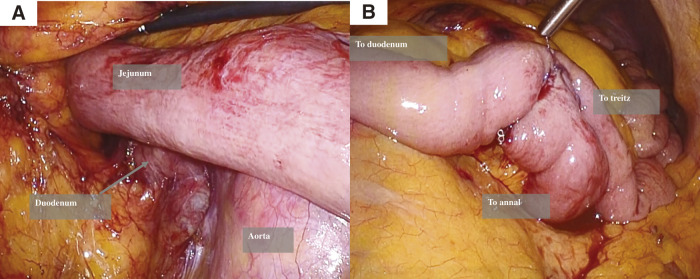
Intraoperative findings. (**A**) Side-to-side anastomosis between the duodenum near the inferior duodenal angle and the lifted jejunum using a linear stapler. (**B**) After construction of the Roux-en-Y limb to prevent BLS. BLS, blind loop syndrome

## DISCUSSION

SMA syndrome was first described as an autopsy finding by Rokitansky in 1861 and later clinically characterized by Wilkie in 1921; therefore, it is also referred to as Wilkie’s syndrome.^3)^ This condition is caused by compression of the horizontal portion of the duodenum between the abdominal aorta and the SMA, resulting in impaired duodenal passage and symptoms of intestinal obstruction, including postprandial epigastric pain, anorexia, bilious vomiting, and weight loss.^4,5)^ SMA syndrome is rare, with a reported prevalence of 0.0024%–0.3%, and predominantly affects young women, who account for approximately 75% of reported cases.^2,6)^ The pathogenesis of SMA syndrome is broadly classified into 2 categories: reduction of mesenteric or retroperitoneal fat tissue and anatomical abnormalities. The former includes severe weight loss and prolonged immobilization, while the latter encompasses congenital anomalies of SMA origin, calcification at the SMA root, and mesenteric traction associated with surgical interventions. These mechanisms share a common feature in that they reduce the aortomesenteric angle and distance, leading to duodenal compression. In contrast, the present case developed duodenal obstruction after EVAR for AAA mimicking SMA syndrome. EVAR is widely used as a minimally invasive treatment for AAA; however, unlike open graft replacement, the aneurysmal sac contents are preserved, and the aneurysm volume does not necessarily decrease immediately. While some reports have demonstrated symptom improvement after EVAR for AAA-associated SMA syndrome due to aneurysmal decompression,^7)^ other studies have described duodenal obstruction caused by transient increases in aneurysm sac pressure or re-expansion related to endoleaks after EVAR.^1)^

Primary compression by the AAA was considered unlikely because symptoms developed 4 years after EVAR. Adhesive obstruction was also excluded due to the absence of prior laparotomy and intraoperative adhesions. Imaging showed compression of the third portion of the duodenum near the residual aneurysm sac, with symptoms arising only after EVAR. These findings suggest that post-EVAR anatomical and mechanical changes were the most plausible cause. Because the aneurysm sac remains after EVAR and contains a stent graft, it may contribute to duodenal compression, while the graft’s stiffness may alter local biomechanics.^8)^ In this case, postprandial increases in intraduodenal pressure likely displaced the duodenum toward the SMA, creating stenosis between the SMA and the stent graft. Thus, the obstruction likely resulted from combined post-EVAR structural changes and dynamic factors rather than from simple aneurysmal compression alone.

In the diagnosis of SMA syndrome, the aortomesenteric angle and aortomesenteric distance are commonly used as supportive imaging parameters,^2)^ with values below 25° and 10 mm, respectively, proposed as diagnostic thresholds.^9)^ However, these parameters should be interpreted as adjunctive findings, and the diagnosis should be based on a comprehensive assessment of clinical symptoms and imaging findings demonstrating duodenal compression. In the present case, the aortomesenteric angle and distance were preserved during asymptomatic periods, whereas during symptomatic episodes, the aortomesenteric distance was markedly reduced to 3 mm and the angle could not be reliably assessed. The patient’s symptoms and imaging findings were consistent with duodenal compression caused by the physical mass effect of the post-EVAR aneurysm sac. Taken together, this case clinically fulfilled the symptomatic features of SMA syndrome; however, the underlying pathophysiology was considered to differ from that of classic SMA syndrome. Specifically, duodenal obstruction was thought to result from combined extrinsic compression caused by the posterior mechanical effect of the stent graft placed after EVAR and anterior compression associated with postprandial gastric distension. Therefore, we diagnosed this condition as “duodenal obstruction after EVAR mimicking SMA syndrome” rather than classical SMA syndrome, based on its distinct mechanism of development. When gastrointestinal obstruction occurs after EVAR, evaluation for endoleaks or aneurysm sac pressurization is essential. In this case, no apparent endoleak was detected, and symptom resolution through additional endovascular intervention alone was considered unlikely. Consequently, a gastrointestinal bypass procedure was selected to address the fixed mechanical obstruction.

Conservative management is generally the first-line treatment for SMA syndrome, aiming to increase retroperitoneal and mesenteric fat through nutritional support and thereby restore the aortomesenteric angle. However, surgical intervention is indicated when conservative treatment fails or symptoms recur.^10)^ Among various surgical options, duodenojejunostomy is widely accepted because of its safety and efficacy. Nevertheless, previous studies have reported that postoperative symptom resolution is incomplete in a substantial proportion of patients.^11)^ Only approximately one-third of patients achieve complete symptom remission, and some develop gastrointestinal motility disorders such as global dysmotility or gastroparesis, occasionally requiring long-term enteral nutrition or total parenteral nutrition. One potential contributing factor is BLS, which results from stagnation of intestinal contents and bacterial overgrowth in surgically created blind segments. BLS has been reported most frequently following side-to-side duodenojejunostomy. In patients with persistent anatomical compression, such as those after EVAR, the risk of BLS may be further increased.

In this case, because the obstruction was caused by sustained mechanical compression after EVAR, conservative nutritional therapy was unlikely to be effective, and a simple bypass alone was considered insufficient due to the risk of BLS. Therefore, a Roux-en-Y duodenojejunostomy was performed. In this case, the jejunum was transected approximately 15 cm distal to the ligament of Treitz to achieve sufficient mobility for a tension-free anastomosis while avoiding excessive redundancy or kinking and securing a stable reconstruction route. To the best of our knowledge, there are no previous reports describing prophylactic Roux-en-Y reconstruction specifically for SMA syndrome or SMA syndrome–like conditions. This approach may reduce the risk of blind loop formation regardless of the underlying mechanism.

## CONCLUSIONS

The procedure was safely completed using a fully laparoscopic approach, and the patient has remained symptom-free during long-term follow-up. In patients with SMA syndrome–like duodenal obstruction caused by fixed mechanical compression after EVAR, laparoscopic duodenojejunostomy with Roux-en-Y reconstruction may represent an effective therapeutic option.

## DECLARATIONS

### Availability of data and materials

The data that support the findings of this study are available from the corresponding author upon reasonable request.
